# The Complexity of the Tumor Microenvironment and Its Role in Acute Lymphoblastic Leukemia: Implications for Therapies

**DOI:** 10.3389/fonc.2021.673506

**Published:** 2021-05-05

**Authors:** Carolina Simioni, Ilaria Conti, Gabriele Varano, Cinzia Brenna, Eva Costanzi, Luca M. Neri

**Affiliations:** ^1^ Department of Life Sciences and Biotechnology, University of Ferrara, Ferrara, Italy; ^2^ Laboratory for Technologies of Advanced Therapies (LTTA) - Electron Microscopy Center, University of Ferrara, Ferrara, Italy; ^3^ Department of Translational Medicine, University of Ferrara, Ferrara, Italy

**Keywords:** microenvironment, ALL, bone marrow, angiogenesis, HIF, hypoxia, therapies

## Abstract

The microenvironment that surrounds a tumor, in addition to the tumor itself, plays an important role in the onset of resistance to molecularly targeted therapies. Cancer cells and their microenvironment interact closely between them by means of a molecular communication that mutually influences their biological characteristics and behavior. Leukemia cells regulate the recruitment, activation and program of the cells of the surrounding microenvironment, including those of the immune system. Studies on the interactions between the bone marrow (BM) microenvironment and Acute Lymphoblastic Leukemia (ALL) cells have opened a scenario of potential therapeutic targets which include cytokines and their receptors, signal transduction networks, and hypoxia-related proteins. Hypoxia also enhances the formation of new blood vessels, and several studies show how angiogenesis could have a key role in the pathogenesis of ALL. Knowledge of the molecular mechanisms underlying tumor-microenvironment communication and angiogenesis could contribute to the early diagnosis of leukemia and to personalized molecular therapies. This article is part of a Special Issue entitled: Innovative Multi-Disciplinary Approaches for Precision Studies in Leukemia edited by Sandra Marmiroli (University of Modena and Reggio Emilia, Modena, Italy) and Xu Huang (University of Glasgow, Glasgow, United Kingdom).

## Introduction

Tumors have a small subpopulation of cells responsible for cancer initiation and relapses (1–3). Leukemic stem cells (LSC), like normal hematopoietic stem cells (HSC), depend on the interaction with the microenvironment or on the niche for their own self-renewal and maintenance.

The microenvironment consists not only of tumor cells but also of physiological tissues which include the stroma (supporting scaffold) with fibroblasts, blood vessels and white blood cells, especially T lymphocytes and macrophages, whose precursors are monocytes deriving from the myeloid lineage. Some tumors have a rich T lymphocyte infiltration (4, 5). Innate immunity cells such as macrophages, granulocytes and immature myeloid cells from which monocytes or macrophages derive are also found in different quantities (6). Today, it is becoming increasingly clear that the immune system can have a double significance, that is, to manifest antitumor activity or to offer a significant contribution to the tumor development, therefore constituting a barrier that opposes the efficacy of anticancer drugs by creating chemoresistance (7). The paradoxical role that the immune system plays in tumor control can be explained by its plasticity that leads to pro or antitumor properties (8–10).

Therefore, considering the role of the tumor microenvironment in neoplastic diseases, it is clear that in the treatment of numerous tumors it is necessary not only to target the tumor cells, but also to control the inflammatory microenvironment that causes resistance to anticancer therapies (11–14). In this regard, clinical studies aim at blocking the recruitment of inflammatory cells into the tumor environment by neutralizing attractive chemokines with specific antibodies, and by taking advantage of suitable strategies to inhibit the activity of cells. A possible antitumor therapeutic strategy therefore is targeting the inflammatory microenvironment in combination with the blocking of immune checkpoints.

In recent years, the interaction of LSC with other cells located in the BM, the so-called medullary microenvironment, has a leading role in these phenomena. In this context, it contributes decisively to the development of the disease and to drug resistance mechanisms and is also decisive in the process that leads stem cells to transform into malignant cells.

In particular, the so-called mesenchymal progenitors, stem cells derived from the BM, constitute a particular population, since they support the niche of both normal and leukemic HSC (15, 16). The rationale is therefore to explore this network of relationships that feeds and protects LSC, in order to develop therapeutic and personalized strategies that could effectively interfere with these communication routes or with the “interlocutors”. In the intertwining of these relationships the process of angiogenesis also represents a crucial aspect, favoring the malignant progression of cancer, both as regards solid tumors and hematological ones (17, 18).

The complexity of interactions within the leukemic microenvironment has been explored for many years. In this review, the aim is to summarize the current understanding of the cellular and molecular network of the tumor microenvironment in ALL, and therefore its ability to modulate the leukemogenesis process, leading to the development of future treatment strategies that can intervene in the remodeling of the leukemic microenvironment.

## Acute Lymphoblastic Leukemia

ALL is one of the most common malignancy in child population (19–22). In pediatric patients, modern protocol therapies are associated with overall success, while the adult population that develops this disease has an unfavorable prognosis: the survival rate in patients from 25 to 59 years old is around 40%, while in the aging the survival rate is usually below 20%.

**Figure 1:**
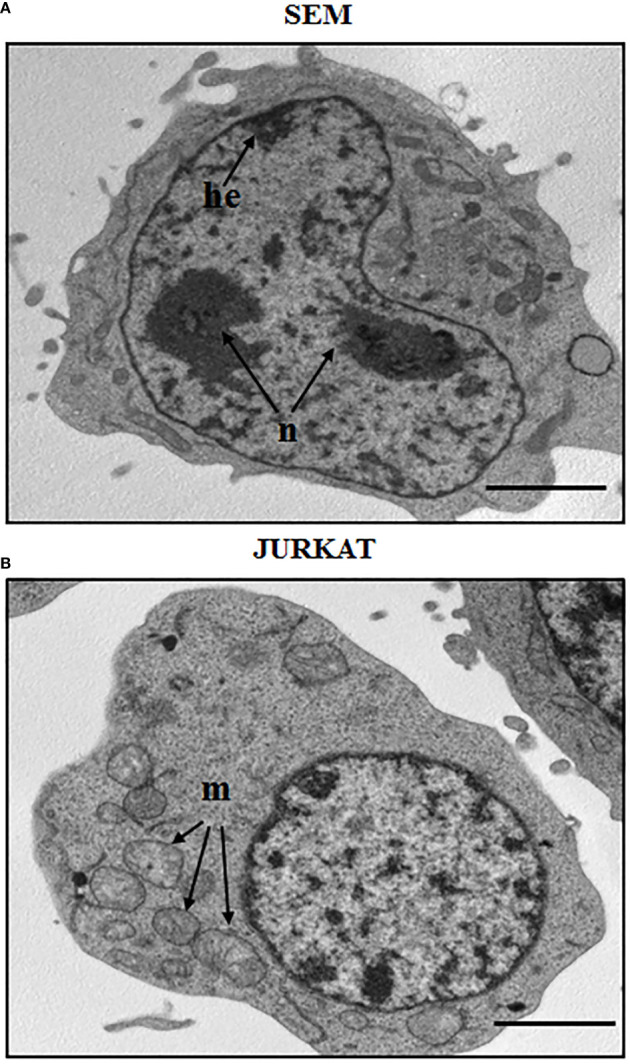
Morphological aspect of a B cell precursor leukemia (SEM) and a T-ALL (Jurkat) cell lines. TEM images of: **(A)** SEM cells at 8000 magnification; **(B)** Jurkat cells at 8000 magnification. n: nucleolus, he: heterochromatin, m: mitochondria. Scale bars correspond to 2 μm. For TEM analysis, samples were fixed in 2.5% glutaraldehyde and post-fixed in 2% osmium tetroxide, dehydrated in acetone solutions and included in Araldite Durcupan ACM (Fluka). Samples were then counterstained with uranyl acetate in saturated solution and lead citrate and observed under transmission electron microscope Zeiss EM910 at 100 Kv.

ALL is more frequent in males, and specifically adolescents and young adults display a greater number of T-cell acute lymphoblastic leukemia (T-ALL) subtype rather than the other ALL progenitors. The B-cell subtype accounts for about 75-80% of ALL cases and it mainly develops in children with a peak incidence of around 2-5 years (23, 24). B-ALL is characterized by an arrest in lymphoid differentiation and therefore in the lymphoid development as pro or pre-B, with recurrent genetic alterations that involve cell cycle and lymphogenesis control genes (21). In adults, prognosis of B-ALL is more consistent at least partially for the BCR-ABL1 fusion protein onset, which is usually found in 2-10% of cases in children. T-ALL subtype is characterized by the accumulation of undifferentiated thymocytes that have acquired multiple genomic aberrations affecting critical transcriptional and signaling networks (25). It represents 12.7% of pediatric cases, and survival rates at 5 years for children and adolescents affected by T-ALL are 70–75%, while in adults the rates are 35–40%. A morphological and structural profile between two ALL cell line subtypes is shown in **Figure 1**, that reports the morphology of a B cell precursor leukemia cell line, SEM, and a T cell leukemia, Jurkat cell line. The images were acquired by Transmission Electron Microscopy (TEM). Observing TEM images, SEM cell nucleus is delimited by a nuclear envelope and contains more than one nucleolus (n) and some areas enriched in heterochromatin (he). Jurkat cells have a well-defined rounded nucleus with envelope, and a larger and more distributed cytoplasm. Around the nucleus, structures such as mitochondria (m) can be appreciated (**Figure 1**).

Chemoresistance and relapse mechanisms are driven by a pool of rare leukemia initiating cells (LICs). These cells are able to escape chemotherapeutic drugs and are mainly involved in self-renewal and in new LICs generation. Moreover, this cellular class has in common several properties with HSCs, that normally leads to the formation of a HSC cell identical to the parent cell, and to a partially differentiated progenitor cell incapable of self-renewal (therefore not stem cells), but with a large replicative potential. The type of differentiation that the progenitor cell undergoes (also called TAC, Transit Amplifying Cell due to its replicative capacity) determines the distinction of blood cells into two large classes: cells from the myeloid lineage and cells of the lymphoid lineage, therefore T lymphocytes, B lymphocytes and Natural Killer (NK) cells (26, 27).

The optimal site for HSC is the BM, that includes specialized areas called niches consisting of different cell types that control the number, quiescence, self-renewal, differentiation and distribution of HSCs (23). The niches are divided into two distinct, but equally connected areas: the first in which the osteoblast plays a primary role (Osteoblastic Niche); the second where endothelial cells predominate and has been called Vascular Niche (23, 28, 29).

In particular, in the vascular niche, sinusoidal endothelial cells favor the survival, proliferation and differentiation of myeloid progenitors and megakaryocytes. HSCs in the Vascular Niche are exposed to hormones, growth factors, oxygen and nutrients, therefore to detect signals and stimuli from the peripheral circulation. This favors self-maintenance, proliferation and/or differentiation (30, 31).

On the contrary, the osteoblastic niche could act as a reservoir since it favors the maintenance of the state of quiescence (32). However, not all the cells of the endosteal surface perform these functions; those that define the osteoblastic niche are represented by the N-cadherin^+^ and CD45^-^ (SNO) subpopulation.

The direct contact between the microenvironment and leukemic cells is therefore essential, and also the activation of different signaling pathways characterizes the microenvironment in a specific way (33, 34). Phosphatidylinositol-3-kinase (PI3K)/AKT-Bcl2 or NOTCH1 appear to be relevant pathways to be induced by microenvironment and leukemic cell connections (15).

Finally, long-term overactivity of reactive oxygen species (ROS) could have deleterious effects on stem cells and on leukemia incidence (35), and in this context several networks are activated in response to ROS increment such as P53, AKT, MAPK and ataxia telangiectasia mutation (ATM).

A better understanding of the contribution of microenvironmental stimuli in mediating leukemogenesis may provide a better scenario of new therapeutic targets in the treatment of leukemias.

### The Role of Cytokines in Leukemic Microenvironment

The inflammatory microenvironment surrounding cancer cells is a complex network in which immune infiltration has long been considered a defense mechanism against malignant tumors. In fact, it can contribute to the development, spread of cancer, metastasis and the onset of resistance to anticancer therapies (36). Cytokines are essential for BM niche homeostasis, and the first studies on B-ALL reported a higher expression of these molecules when compared with healthy BM samples, suggesting an autocrine/paracrine regulation of leukemic cells and cytokines (37). Among the cytokines with more modulatory activity IL-7, IL-8 and IL-15 are the most involved.

**Figure 2:**
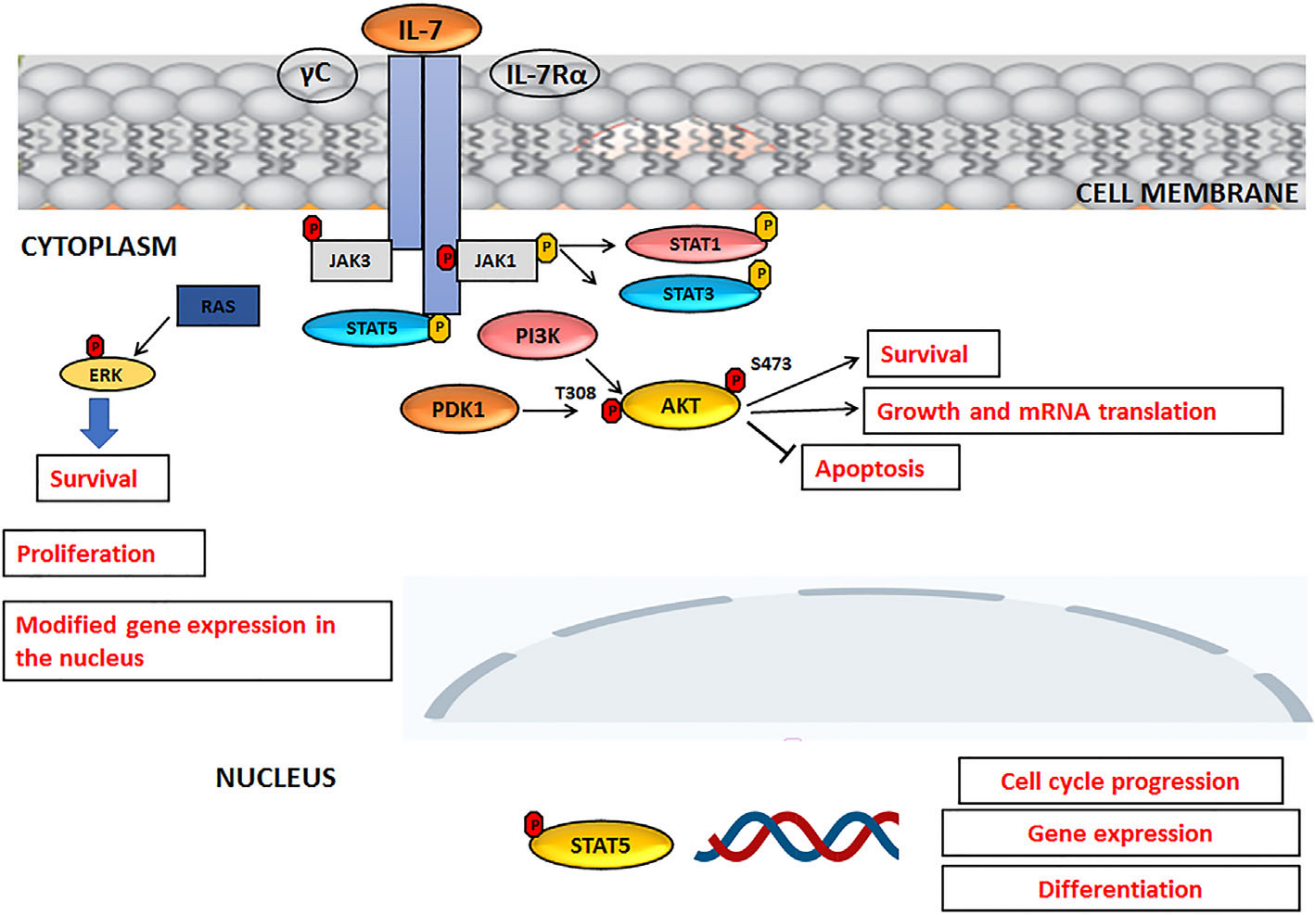
IL-7 signaling pathway. For details, please see the text.

Interleukin-7 (IL-7) is a cytokine produced by stromal cells and promotes hematological malignancy development and differentiation (ALL, T-cell lymphoma). It functions primarily as a growth factor and an antiapoptotic molecule, essential for the differentiation of T lymphocytes with TCR γ− δ. It also works as a cofactor during myelopoiesis and is capable of activating monocytes/macrophages and NK lymphocytes. Its receptor is a heterodimer consisting of an alpha chain, which binds IL-7, and a gamma chain, a common component of the IL-2, IL-4, IL-9, IL-15 receptor. Experimental data suggest that, even in humans, IL-7 could act as a factor stimulating leukopoiesis, given that it is of great importance in the treatment of bone marrow transplant patients (38). Like normal T and B progenitors, most T-ALL and B-ALL cells express functional receptors for IL-7 (IL-7R) which are able to promote leukemia survival and proliferation both *in vitro* and in patient- derived xenograft assays, suggesting a role for IL-7R/IL-7 pathway in ALL progression. IL-7 and IL-7R activate three main signaling networks: STAT5, PI3K/AKT/mTOR and MEK/ERK, leading to leukemia cell progression and survival, also with regard to T-ALL (25, 39, 40). An overview of the IL-7 signaling, upon activation IL-7R, is summarized in **Figure 2**. Upon IL-7 binding, IL-7 receptor α chain (IL-7Rα) and γ-common (γc) chains dimerize, with consequent activation of JAK3 and JAK1 kinases that bind to the intracellular domain of γc and IL-7Rα, respectively. JAK1 phosphorylates the IL-7Rα intracellular domain with activation of PI3K and STAT, resulting in STAT translocation to the nucleus and in the transcription of target genes involved in cell cycle progression or differentiation. Phosphorylated AKT promotes cell survival through degradation of pro-apoptotic proteins and glucose uptake (**Figure 2**).

The activity of IL-7 in supporting ALL development was firstly documented in *in vitro* models (41, 42). In has been shown that B-ALL primary cells co-cultured with stromal cells displayed a CXCL12-dependent proliferation which was concomitant to the presence of IL-7 and, to a lesser extent, to the expression of IL-3 (43). This synergistic/additive effect was partially correlated with the increased phosphorylation of components of CXCL12-activated signaling networks (e.g., PI3K/AKT and MEK/ERK). In *in vivo* models, mice with IL-7 receptor knocked out exhibited thymic atrophy, developmental arrest of double-stage positive T cells, and severe lymphopenia. Administration of IL-7 in mice leads to an increase in B and T cells and in T cell recovery after cyclophosphamide addition or after BM transplantation (44). Thymic epithelial cells increased survival of T-ALL primary cells through IL-7, suggesting a functional role for the thymic microenvironment in the acquisition of selective growth advantage of leukemic cells (23). Importantly, in BM stromal or thymic epithelial cells co-cultured with T-ALL cells enhanced survival specifically required IL-7/IL-7R interactions, while the blocking of either IL-7 or IL-7R significantly reduced apoptosis inhibition mediated by the leukemic microenvironment (45). Moreover, IL-7 receptor is upregulated in about 50% of T-ALL through mutational activation of NOTCH, considered an upstream regulator (42).

It has also been shown that C-C Motif Chemokine Ligand 2 (CCL2) and IL-8 were able to increase the adhesion of ALL cells to BM stromal cells, in a stromal-dependent manner, and to promote survival and proliferation of BM stromal cells, while they seem not to have effect on most precursor B-cell ALL survival or migration. Indeed, it was found that the BM plasma levels of CCL2 and IL-8 in ALL children at diagnosis were significantly higher than in healthy controls. Moreover, in T-ALL IL-8 production is physiologically regulated by a specific signaling network, represented by CXCL12/CXCR4 axis and the nuclear factor-κB (NF- κB) and JNK/AP-1 pathways. Therefore, the analysis of the molecular mechanisms that intervene in the modulation of IL-8 could be important to further hypothesize possible alternative therapeutic strategies (46, 47).

The role of IL-15 has been reported in a xenograft model of human B cell precursor (BCP)-ALL in immunocompromised mice, characterized by infiltration of ALL blasts into the CNS. Blasts and brain resident cells induced aberrant activation of host cells in the brain microenvironment, with a consequent cytokine and exosomes release, including IL-15 that binds to astrocytes and brain vessel endothelial cells. It has been shown that the silenced expression of either IL-15 in the NALM6 cell line significantly decreased CNS infiltration in engrafted mice, thus providing important points by which lymphoblasts modulate the brain microenvironment (48).

### The Role of Signaling Networks in Leukemic Microenvironment

LICs and BM niches are characterized by specific signal transduction pathways which, if expressed in an aberrant way, influence the mechanisms of survival, proliferation, drug resistance and cellular invasion. Therefore, the aim is to identify the molecular mechanisms that promote and support tumor proliferation and downstream survival of important signaling pathways critical for the onset and progression of ALL. Two important signaling networks are CXCL12-CXCR4 and NOTCH.

#### CXCL12-CXCR4 Network

The activation of the CXCR4 receptor by the chemokine CXCL12 (also known Stromal cell-Derived Factor-1, SDF-1) represents one of the most studied transductional axes, in the modulation of the HSC migratory behavior and in the localization of the leukemic cells to their niches (49, 50). As a demonstration of the important biological function performed by CXCL12, the amino acid sequence of this chemokine is extremely conserved among the different species during evolution. CXCL12 plays a role of primary importance already during embryonic development; experimental mouse models of gene knockout for CXCL12 or its CXCR4 receptor showed defects in the heart, intestines, circulatory and nervous systems, which are already lethal in the early stages of development (51). Furthermore, the lack of ability of the hematopoietic progenitors to colonize the bone marrow is significant, and this phenomenon can be related to the role of CXCL12 in regulating the migration of HSC and their trafficking between the different hematopoietic niches. The mechanisms that regulate the activity of CXCL12 remain largely still to be characterized.

It is currently believed that the expression of CXCL12, as well as the CXCR4 receptor, is extremely dynamic and regulated by mechanisms of autocrine and paracrine secretion (52). In particular, CXCR4 is widely expressed within the bone marrow by immature or maturing hematopoietic cells, of which it finely regulates the processes of homing, bone marrow retention or migration and entry into the circulation (53). Worthy of note is also the high expression of both CXCL12 and CXCR4 by stromal cells and medullary endothelial cells, which seems to confirm the existence of autocrine and paracrine stimulation circuits within the medullary microenvironment (54).

The activation of CXCR4 induces the up-regulation of different signaling networks such as p38 mitogen-activated protein kinase (p38 MAPK), MEK/ERK, PI3K/AKT, and protein kinase C (PKC) (50). It has been reported that PI3K/AKT network could control cell cycle progression by regulating the cyclin B1 and stathmin expression/phosphorylation (55). Moreover, both MEK/ERK and p38 MAPK kinase promoted T-ALL migration through interactions with α2β1 integrin, suggesting that this blockade could be a valuable target for T-ALL therapeutic treatment (56).

CXCL12 and CXCR4 could be also important in B-ALL. Indeed, an elevated expression of CXCR4 in B-ALL blasts has been related to a worse patient outcome, and the expression of CXCR4 active form has been associated with a poor survival for B-ALL patients (23, 57). Further evidence emerged from studies conducted on a CXCR4 receptor antagonist, AMD3100 (Plerixafor). It was reported that a single injection of AMD3100 is able to induce rapid mobilization of hematopoietic progenitors in mouse models, as well as in humans (58). The molecular mechanism underlying this effect could be the binding of the antagonist to the receptor expressed by stem cells and hematopoietic progenitors, or to the receptor present on the surface of stromal cells, or both. In particular, it was seen that AMD3100 was able to block chemotaxis both in the B-ALL cells NALM6 and in primary B-ALL cells, therefore enhancing the effects of cytokines and growth factors found in the BM microenvironment (59).

CXCR4 is also highly expressed in T-ALL cells (60), reporting also CXCR7 as the second receptor for CXCL12 that is able to augment CXCR4 response in ALL, therefore contributing to leukemia maintenance by initiating cell recruitment to BM niches (60). A genetic targeting of CXCR4 in mouse T-ALL after disease onset could lead to a rapid, sustained disease remission, and CXCR4 antagonism is able to block human T-ALL in primary xenografts. Loss of CXCR4 decreased leukemia initiating cell activity *in vivo* and modulated targeted key T-ALL regulators, such as the MYC pathway (61).

It was also found that BM stromal cells are able to up-regulate IL-8 mRNA in T-ALL cells through the activity of CXCR4, and exogenous CXCL12 induced IL-8 mRNA synthesis in primary T-ALL cells, with consequent activation of nuclear factor –κB (NF- κB) and c-Jun (47).

#### NOTCH Network

NOTCH pathway is one of the most evolutionarily conserved signaling cascade across multiple species and it is involved in the regulation of essential cellular functions, from cell fate decision during embryo development, to the regulation of cell proliferation, cell growth and survival (62, 63). NOTCH is a surface receptor protein and binding of its cognate ligands, such as Jagged 1 and 2 (JAG1-2) or Delta-Like 1-4 (DLL1-4), promotes the proteolytic cleavages of the extracellular domain by the Tumor Necrosis Factor-Alpha Converting Enzyme metalloproteinase (TACE), followed by the cleavage mediated by a γ-secretase which facilitates the release and nuclear translocation of the NOTCH intracellular domain (ICN1), where it recruits coactivators to form a transcription-activating complex, finally mediating its biological functions (64). Importantly, the activity of NOTCH family members is context-dependent, exerting sometimes even opposite roles (promoting cell growth or apoptosis) in different cell types and/or depending on the expression of specific transcriptional programs. Considering its pleiotropic activities and the spectrum of cellular processes in which it is involved, aberrant expression/activity of NOTCH signaling components has been associated with a variety of cancers, from solid tumors to hematological malignancies where, given its dependency on the cellular context (genetic and/or molecular variabilities), NOTCH can act either as an oncogene or as a tumor-suppressor (65, 66). Gain-of-function mutations as well as non-mutational hyperactivation of NOTCH1 and NOTCH2 have been described in B-cell specific lymphoproliferative disorders, including Chronic Lymphocytic Leukemia (CLL) and different Non-Hodgkin lymphoma subtypes (i.e., Diffuse Large B cell lymphomas, Mantle Cell lymphoma, Burkitt’s lymphoma) (67). NOTCH1 mutations represent one of the most frequent genetic modifications in T-ALL, often in association with mutations/activation of the Hedgehog signaling pathway, suggesting cooperation between those pathways, even though this genetic interaction has not been described yet. In both CLL and T-ALL, interference with NOTCH activity has been shown to have a negative impact on the growth of cancer cells, confirming the oncogenic potential of NOTCH in those diseases (68). Whether NOTCH plays a similar role in B-ALL is still a matter of debate. Several studies suggested a contribution of genes involved in developmental signaling pathways, i.e., Hedgehog and NOTCH, in promoting survival and chemoresistance of leukemic cells, including B-ALL (69–71). Additionally, there are evidence showing that inhibition of NOTCH activity in B-ALL cell lines results in enhanced sensitivity to cytotoxic treatments, suggesting protumoral properties for NOTCH in B-ALL. However, conflicting data suggest that NOTCH activation can be deleterious for leukemic cells, as enforced expression of NOTCH in human or mouse B-ALL cells results in growth arrest and apoptosis (72). This is in line with a study reporting the activity of NOTCH in inducing apoptosis in several B-ALL cell lines: here, the activation of NOTCH resulting in NOTCH target Hairy/Enhancer of Split1 (HES1)-mediated PARP1 activation and subsequent apoptosis-inducing factor (AIF)-induced apoptosis may in part explain the contrasting effects of NOTCH signaling in B-ALL versus T-ALL, representing a mechanism of cell-specific consequences of NOTCH activation. In a recent work, the characterization of a new *in vitro* B-ALL model has been proposed. The model is called VR-ALL and is a common-type B-ALL cell line with mutations in some components of NOTCH signaling, including NOTCH1 and NOTCH2. This mutated cell line gives rise, once injected into immunodeficient NOG mice, to a mouse xenograft model of B-ALL. VR-ALL has reported to be sensitive to γ-secretase inhibitors (GSIs) as well as conventional anti-leukemic drugs, therefore helping to deepen further the role of NOTCH signaling in B-ALL (71). NOTCH activity is highly contextual and thus, it can be significantly influenced by the genetic landscape and/or the transcriptional program characterizing the cellular system used in the experimental settings. In this view, the use of genetically and biologically distinct cell lines might explain, at least in part, the discrepancy between the different studies. Importantly, several inhibitors of NOTCH pathway have been developed and entered clinical trials for the treatment of CLL and T-ALL and could be rapidly extended for the treatment of aggressive B-ALL, if supported by consistent pre-clinical evidence.

Making a connection between the IL-7 signaling pathway and NOTCH1 in the characterization of the leukemic tumor microenvironment, another important and aberrant signaling pathway is worthy of note, and it is represented by PI3K/AKT/mTOR. Overactivation of the PI3K/AKT/mTOR signaling pathway is a common occurrence in many patients with ALL, portends a poor prognosis and for many years literature highlighted that this network, constitutively active in ALL, increases cell proliferation, survival and drug resistance (22, 25, 73–77). The activation of the PI3K/AKT/mTOR signaling pathway also results from the close correlation between the inputs deriving from IL7Rα/JAK signaling, NOTCH1-mediated upregulation of IL7Rα, NOTCH1 and the modulation of specific microRNAs (miRNAs) that induce the inhibition of PTEN tumor suppressor gene expression, reduction of PTEN induced by phosphorylation of casein kinase 2 (78), inactivation of phosphatase SHIP1, or elevated ROS concentration (76).

## The Role of Angiogenesis in ALL

Many tumors have a noticeable increase in vessel density, which causes the tumor to transition from benign to malignant, in a process referred to as “angiogenic switch”. The formation of new vessels is a complex process involving many types of cells including macrophages. In fact, the overexpression of colony stimulating factor 1 (CSF-1), in a mouse model, resulted in a premature accumulation of macrophages in the hyperplastic lesion, an early angiogenic switch and consequently accelerated the transition to malignancy (79).

The angiogenesis process in hematological malignancies is similar to that reported in solid tumors (80).

During tumor development and progression, cancer and stromal cells often are in a poor oxygen condition and have poor access to nutrients. Indeed, in addition to the cellular components, other physiological factors regulate the stem cell niche. A physiological modulator of particular interest is hypoxia (17, 81). Some studies suggest that hypoxia allows for a series of events in the tumor microenvironment that lead to the expansion of aggressive clones from heterogeneous tumor cells and promote a lethal phenotype. The hypoxic response is mainly attributed to hypoxia inducible factors (HIFs).

### HIF Signaling

**Figure 3:**
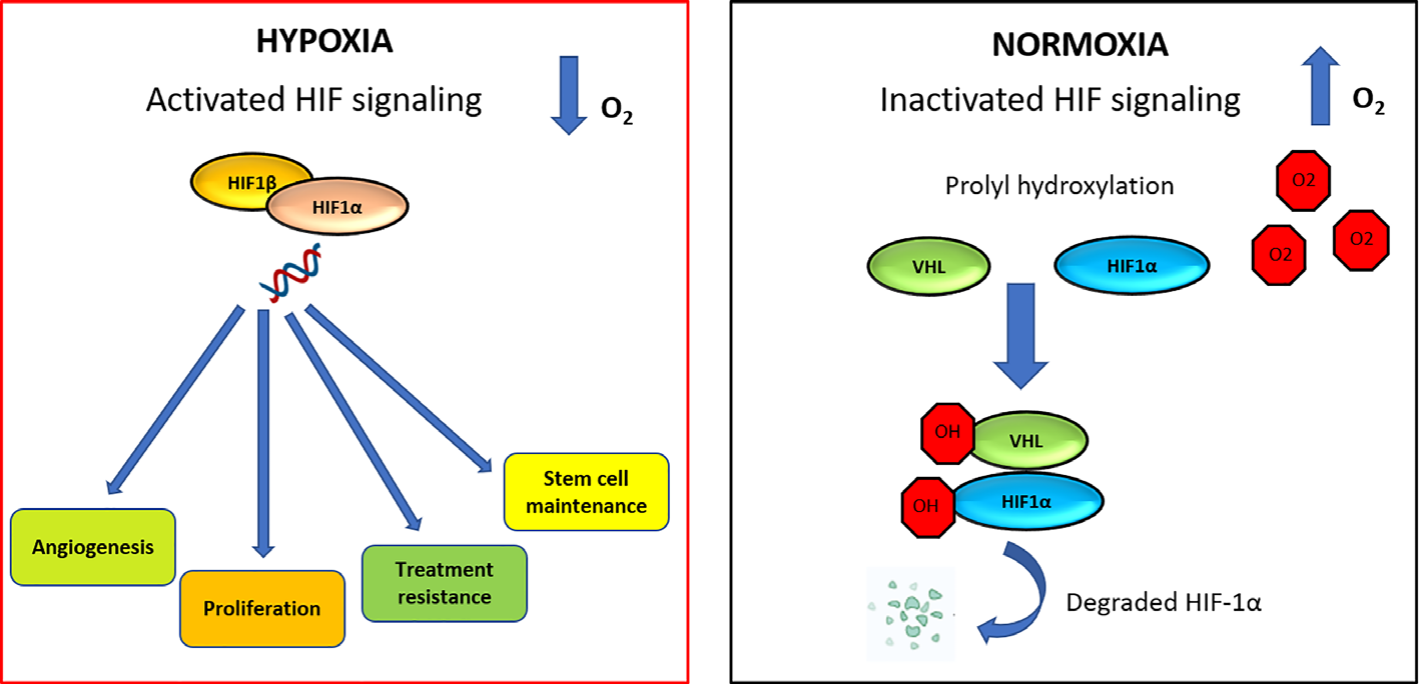
HIF signaling network in normoxic and hypoxic conditions. In hypoxia condition, HIF-1α is able to increase angiogenesis process, proliferation, therapy resistance mechanisms and stem cell maintenance. Under normoxic condition, VHL is the recognition component of an E3 ubiquitin-protein-ligase, target for the proteasomal degradation of HIF-1α. As a result, HIF-1α is modified and it is recognized by a proteasome and rapidly degraded.

HIF-dependent signaling can induce changes that promote cancer progression, survival, stem cell maintenance and vascular remodeling (82, 83). The HIF family of transcription factors includes HIF1, HIF2, and HIF3. These factors all have a HIF-α subunit, that is extremely sensitive to oxygen (HIF1-α, HIF2-α, or HIF3-α, respectively). Each of these subunits contains two proline residues (HIF1-α: P402/P564 and HIF2-α: P405/P531), which are hydroxylated in normoxia condition by proteins containing prolylhydroxylase (PHD). Hydroxylation of proline residues promotes binding to the von Hippel-Lindau tumor suppressor (pVHL), which leads to ubiquitination and degradation of HIF1-α (**Figure 3**) (84). High expression of HIF-α was detected in 67% of patients with acute myelogenous leukemia (AML) (85), 66.7% of patients with pediatric ALL (86) and in 100% of patients with B-CLL patients (87).

The importance of HIF-1α in leukemia microenvironment is given by the fact that it has an important role in differentiation and growth of stem cells, maintaining also the ability of HSC to regulate ROS and to keep a quiescence state (35). It was demonstrated that in T-ALL NOTCH1 is required for HIF-1α stabilization, contributing to HIF-1α- dependent proliferation, invasion and chemoresistance (88). Moreover, it has been reported that in Jurkat cells HIF-1α could be significantly regulated by a protein, called seven in absentia homolog 2 (SIAH2), involved in invasion and metastasis processes (89).

In T-ALL Jurkat cells, SIAH2 knockdown led to increased apoptosis and decreased proliferation. Moreover, PHD, P27 and Caspase3 were upregulated and HIF-1α, Vascular Endothelial Growth Factor (VEGF), VEGF Receptor 2 (VEGFR2), Matrix Metallopeptidase 13 (MMP-13), CyclinE1, C-myc and BCL2 were downregulated in the same cell model, making SIAH2 an attractive therapeutic target, also in correlation with HIF-1α modulation (89). In another work β-catenin transcription was reported to be upregulated by hypoxia HIF-1α stabilization, and deletion of the same transcription factor was able to reduce LIC frequency. Of note, the deletion of β-catenin or HIF-1α did not impair the growth or viability of bulk tumor cells, suggesting that components of the Wnt pathway and HIF specifically support leukemia stem cells (90). HIF-1α is also correlated to mTOR signaling, frequently aberrant in ALL models: coculture of ALL with stromal cells in hypoxic condition was able to induce HIF-1α and AKT/mTOR networks, and mTOR inhibition decreased HIF-1α, inducing ALL cell chemosensitization (91). HIF-1α expression has also been reported to be associated with worse overall and event-free survival in a Ph-negative pre-B ALL model, implicating that inhibition of AKT signaling or blockade of HIF-1α-mediated pro-survival signaling events may improve clinical outcomes in pre-B ALL (91). However, further studies are certainly needed to clarify HIF oncogenic and/or tumor suppressor activity, in particular in the hematological cancer stem cells (HCSCs).

Therefore, it appears extremely interesting to target hypoxia mechanism, the tumor tissue and the microenvironment, mainly due to the point that hypoxia leads to resistance to anticancer therapies. Some of the therapy-resistance mechanisms include (i) increased expression of drug efflux pumps (such as P-glycoprotein, Pgp), (ii) oxygen-dependent cytotoxicity of some molecules, (iii) mutations conferring resistance, (iv) selection of insensitive cells to p53-mediated apoptosis and (v) decreased cell proliferation rate in hypoxic regions (92).

### VEGF and Thrombospondin-1 (TSP-1)

In addition to HIF-1α, numerous other angiogenic regulators in the hypoxic environment are signal proteins and bind to stimulatory or inhibitory receptors located on the surface of vascular endothelial cells. The VEGF-A and TSP-1 are well known angiogenesis modulators: the first one is an inducer, while the latter is an angiogenesis inhibitor. The VEGF-A gene encodes ligands that are involved in governing the growth of new blood vessels during embryonic and postnatal development, and then in the adult in regulating the survival of endothelial cells and neoangiogenesis (93). Three tyrosine kinase receptors (VEGFR-1 -2 -3) regulate this signal pathway at several levels, demonstrating its complexity. Therefore, the expression of the VEGF gene can be hyper-regulated both by hypoxia and by oncogenes signals (94). Moreover, VEGF ligands can be sequestered in the extracellular matrix in latent form, and be subjected to release and activation by a protease degrading the extracellular matrix (e.g., MMP-9) (95).

VEGFR-1 and -2 are mostly expressed on vascular endothelial cells, and the activation of VEGFR-2 is predominantly responsible for mediating VEGF-dependent angiogenesis and the induction of vascular permeability (96). VEGFR-1 is also expressed on HSCs and leukemic cells, vascular smooth muscle cells, different solid tumors such as colon cancer, and in monocytes (97, 98), while VEGFR-2 is expressed on endothelial progenitor cells and megakaryocytes (99). VEGFR-3 is largely restricted to lymphatic endothelial cells (100) and was analyzed in the plasma concentrations in comparison and in combination with CA 15-3 in some cancers in relation to the control groups, to better define its role as a tumor marker (101). The first finding that leukemia progression was accompanied by increased bone marrow vascularization was provided by Judah Folkman’s and collaborators (102) who demonstrated that the BM of ALL patients had a higher number of blood vessels compared with controls, and this was assessed immunohistochemically by factor VIII staining of bone marrow biopsies. Moreover, VEGF and basic fibroblast growth factor (bFGF) were also detected in urine and peripheral blood samples from ALL patients and correlated with the increase in bone marrow angiogenesis (103). VEGF can be regulated by signaling pathways such as PI3K/AKT, as demonstrated in B-ALL cell lines and patients, where an overexpression of heme oxygenase-1 (HO-1) was found in the bone marrow stromal cells (BMSCs) (104). It was found that B-ALL cell lines became resistant to the chemotherapeutic agent vincristine (VCR) in presence of VEGF recombinant protein induced by HO-1 expression. Therefore, VEGF may promote VCR resistance in B-ALL cells, and this mechanism is stimulated by PI3K/AKT network.

On the other hand, TSP-1 exerts an anti-angiogenic activity and could be important in the maintenance therapy of ALL patients, whose effect is anti-endothelial. In ALL children in the maintenance phase consisting in daily oral mercaptopurine, weekly oral methotrexate and VCR once a month for 12 months and dexamethasone for 5 days/month for 12 months, circulating endothelial cells (CEC), endothelial progenitor cells (EPC) and endothelial microparticles (EMP), but also VEGF, VEGFR-1 and Ang-2, TSP-1 and regulatory T lymphocytes (Treg) were monitored. It was reported a statistically significant decrease in EPC and EMP counts throughout the maintenance phase associated with a significant increase in TSP-1 levels, therefore pointing out an antitumor maintenance therapy activity with involvement of anti-angiogenic effects (105). TSP-1 and other epi-driver genes such as LYN, TRAF3 or FLT1 were also analyzed in B-ALL cells and in patients, underlining their use as prospective biomarkers in ALL progression or as targets for innovative therapeutic agents that can be involved in altered DNA methylation and gene expression (106).

## Therapeutic Options Targeting the Microenvironment in ALL: Focus on Some Recent Therapies

As mentioned in the previous sections, leukemia cell microenvironment involves several factors and processes including expression of adhesion molecules, interactions between cancer and stromal cells, cytokine and growth factors, and angiogenesis. It is therefore extremely important to adopt and at the same time to find innovative therapeutic strategies that can slow down, at least in part, the progression of ALL, the cell-cell interactions at the level of the tumor microenvironment and the angiogenesis process, which as previously mentioned, is intrinsically involved in the characterization of the leukemic microenvironment, in the development of stem cells but above all in the scaffolding of the entire vascular network that determines the profile of the neoplasm. Consequently, it appears to be relevant to block the interactions between malignant cells and their niche, and to identify innovative and selective Small Molecule Inhibitors (SMIs) able to inhibit most of the components forming part of the signaling pathways that affect the niche and the BM directly.

In the following sections the most recent evidences regarding the inhibitory activity of some classes of compounds at the level of the leukemic microenvironment are shown. These drugs are also summarized in **Table 1**.

**Table 1:** A summary of the most recent evidences of drugs targeting the microenvironment in ALL.

Drug	Relevant effects	Clinical Trials	Reference(s)
**Prodrugs**			
PR104	Hypoxia condition: hypoxia-selective cytotoxicity *via* DNA crosslinking	–	(107–109)
Nelarabine	Normoxia condition:	NCT00501826	(110–117)
	Cell growth blockage	NCT03020030	
	ALL sensitization improvement to other drugs (AraG)		
OBI-3424	Cytotoxic activity	NCT04315324	(118)
	Prolongation the event-free survival		
	Disease regression		
**HIF inhibitors**			
Echinomycin	Improvement of the survival of mouse models	–	(119)
	Cell growth blockage		
**Proteasome inhibitors**			
Bortezomib (Velcade™)	Apoptosis induction Activation of caspase-3, -8 and -9	–	(126–133)
	Angiogenesis pathway inhibition		
	Stimulation of HDAC inhibitors activity		
	Alteration of mitochondrial transmembrane potential		
Carfilzomib	Apoptosis and autophagy induction		(134–137)
	Minimal Residue Disease response improvement		

### Prodrugs

A prodrug is commonly defined as a substance that is inactive in its initial form. Once administered, the compound turns into an active state (138). The drug then takes effect to treat a specific disorder or condition at a desired time interval. Metabolization changes the molecular structure of a prodrug and this process divides this drug substance into one in which the molecules break down into fragments and into another that becomes soluble after being metabolized.

Remaining in the context of the normoxic or hypoxic condition, a hypoxia activated prodrug (HAP) is represented by PR104, that is a phosphate ester, rapidly hydrolyzed *in vivo* to the corresponding alcohol, PR104A, therefore acting as a HAP. In hypoxia condition, PR104 is reduced to the amines PR104H and PR104M, which induce DNA cross-linking in hypoxic cells (107). PR104, that could be activated also by the enzyme aldo-keto reductase 1C3 (AKR1C3) showed remarkable activity in *in vivo* B-ALL mouse models, suggesting that sensitivity is correlated with AKR1C3, whose expression could be used as a biomarker to select patients for this treatment in future clinical trials (108). In detail, an important aspect highlighted by this study was the great expansion of the hypoxic zones in the BM of leukemic mice, with a consequent loss of vessels integrity with increased leukemic cell burden. BM samples from B-ALL patients, immunostained for HIF1α expressed also in stromal cells, showed a strong positivity at diagnosis, which was impressively reduced when patients achieved a remission (108). The efficacy of PR104 has been reported also in preclinical models of pediatric T-ALL (109).

Several studies are ongoing to generate new HAPs that could be stimulated and activated by hypoxia condition for new molecularly targeted inhibitors having potential benefits in ALL treatments.

As regards instead the non-hypoxic prodrugs, one example is Nelarabine, a water-soluble prodrug of 9-β-Darabinofuranosylguanine (ara-G), a purine nucleoside analogue preferentially accumulating in T-lymphoblasts (110). Nelarabine is indicated for the treatment of patients with T-ALL (111–113) and T-cell lymphoblastic lymphoma (T-LBL) (115) that have not responded or have relapsed after treatment with at least two chemotherapy regimens.

Focusing more in detail on clinical trials currently active, Nelarabine was recently well tolerated in combination with the chemotherapy regimen Hyper-CVAD in the treatment for T-ALL (115), and its activity is involved significantly in the modulation of different signaling networks, mainly by targeting aberrant PI3K/AKT/mTOR signaling pathway (111, 116). Phase II recruiting study of effects of Hyper-CVAD and other drugs with Nelarabine in previously untreated T-ALL and Lymphoblastic Lymphoma is also active (please see www.clinicaltrials.gov, Identifier: NCT00501826). This Interventional Clinical Trial involves the enrollment of approximately 160 participants, and the crucial objectives of the study include the determination of complete remission (CR) following treatment, the safety and overall survival of previously untreated patients with T-cell ALL and T- cell lymphoblastic lymphoma, as well as the efficacy of two other antineoplastic drugs, pegaspargase and venetoclax. Nelarabine would be administered for several days and pegaspargase in multiple treatment steps. The study is recruiting and a further objective foresees how the combination of different chemotherapeutic agents can influence the different phases of cell growth, blocking it as much as possible, also influencing protein synthesis in terms of inhibition, considering the role of pegaspargase. Pegaspargase and Nelarabine are included in treatments with other chemotherapeutic agents also in newly diagnosed ALL children and adolescents (please see www.clinicaltrials.gov, Identifier: NCT03020030).

In a recent study, a combined pharmacogenomics data combined with data from an ALL cell line panel and patients reported the sensitivity differences between T-ALL and B-ALL. It was seen that the depletion of the deoxynucleotide triphosphate (dNTP) hydrolase SAMHD1, that is able to cleave and inactivate AraG, sensitized ALL cells to AraG, underlining the role as a therapeutic target to improve nelarabine therapies in ALL patients (117).

Recently, a novel AKR1C3-prodrug, OBI-3424, has shown significant antitumor activities in preclinical models of pediatric T-ALL, in relapsed/refractory T-ALL and also in solid tumors (118). A relevant reduction in BM infiltration was observed in ALL patient-derived xenografts (PDX) characterized by aggressive and fatal disease (118). Toxicity of OBI-3424, as well as the evaluation of the complete remission (CR), the event-free survival (EFS), the relapse-free survival (RFS) and the overall survival (OS) in patients with relapsed/refractory T-ALL are also the objectives reported in a phase II recruiting trial (please see www.clinicaltrials.gov, Identifier: NCT04315324).

### Targeting HIF

Targeting HIF-1α represents another promising approach to block hypoxia-dependent progression, cell growth and drug resistance (92).

Although HIF represents an excellent and promising target in the characterization of the leukemic tumor microenvironment, there are currently no recent drugs capable of selectively inhibiting this transcription factor in ALL. The goal is therefore to develop more specific SMIs in ALL in order to make a significant breakthrough in the reduction of angiogenesis. In previous studies, echinomycin inhibiting HIF activity in lymphoma and leukemic stem cell models has been demonstrated, significantly improving the survival of mouse models and limiting the progression and growth of cancer cells (119). A very recent study instead reports the possible correlation between the activity of this transcription factor and the signaling pathway of PI3K/AKT/mTOR (120). In fact, it has been shown that in T-ALL cells the mTOR inhibitor rapamycin in normoxia is able to mimic the effects of the hypoxic condition, decreasing cell growth and increasing quiescence. Knocking down (KD) HIF-1α, a key regulator of the cellular response to hypoxia, the effects observed in hypoxic T-ALL were antagonized with a return to chemo-sensitivity. Therefore, the inhibition of mTOR in HIF1α KD T-ALL protected leukemic cells from chemotherapy. This mechanism may help to suppress the drug resistance of T-ALL in hypoxia, suggesting new therapeutic protocols (120).

### Proteasome Inhibitors

The potential effects of proteasome inhibitors as anticancer therapy as well as for the treatment of other diseases have long been studied (121–123). Studies have shown that these agents could induce programmed death in leukemia cell lines, Burkitt’s lymphoma cell and other blood cancers and solid tumors (122, 124, 125).

Proteasome inhibitors are a relatively new class of cancer-targeted therapy. An example is Bortezomib (Velcade™) that is known to induce apoptosis, blockage of cytokine effects of myeloma and also on ALL models (126–129). Bortezomib works by activating two upward activating kinases of NFκB (RIP2 and IKKβ) and causes non-proteasomal degradation of IκB and stimulates DNA binding of NFκB (130). Moreover, it acts at the medullary level by stimulating the osteoblastogenetic process and inhibiting the angiogenic pathway (127).

The most recent studies reported how this drug could be effective in ALL relapsed and refractory pediatric patients, in addition with reinduction chemotherapy based on combinations with mitoxantrone, vincristine, pegaspargase or dexamethasone (131). Response rates were most significant in T-ALL. Notably, no patient in the study demonstrated neurological toxicities, despite this may represent a side effect induced by bortezomib, together with pulmonary complications. In ALL models, Bortezomib is able to enhance the activity of HDAC inhibitors, enzymes that normally regulate the structure and function of chromatin (132). In detail, treatment of pre-B ALL cells with the HDAC inhibitor panobinostat influences cell viability, induces apoptosis at increasing time treatment and leads to an increase in the expression levels of cyclin-dependent kinase inhibitors p21 and p27, correlating with the reduction of c-Myc and CDK4 mRNA expression level. Apoptotic cell death through the alteration of apoptosis-related genes is further enhanced by administration to cells with Bortezomib. Indeed, this drug led to an enhanced cytotoxicity of the HDAC inhibitor through decreasing the mRNA expression levels of anti-apoptotic target genes (132).

In Jurkat and Molt-4 T-ALL cells, the co-treatment of bortezomib with the glycosidic antibiotic daunorubicin enhanced the activation of caspase-3, -8 and -9, and the effect was reversed by the pan-caspase inhibitor, Z-VAD-FMK (133). Moreover, cotreatment with bortezomib and daunorubicin enhanced the collapse of mitochondrial transmembrane potential and upregulated the proapoptotic protein, B-cell lymphoma 2 (Bcl-2)-interacting mediator of cell death (Bim). These findings can help to deepen preclinical and clinical investigations (133).

The activity of Bortezomib was recently compared with a second recent proteasome inhibitor, Carfilzomib, whose efficacy was reported both in B and T-ALL models (134, 135). It was in fact seen that Carfilzomib showed relevant activity in the majority of ALL cell lines except for the P-glycoprotein-positive t (17, 19) ALL cell lines, and the knockout of the IKZF1 gene was associated with a favorable response to Carfilzomib treatment (136), making the association of Carfilzomib with the current chemotherapy protocols a new therapeutic option for refractory ALL with P-glycoprotein-negative leukemia cells. In a Phase I study, increasing doses of Carfilzomib were associated with Hyper CVAD in Patients with Newly Diagnosed ALL, showing promising results, also in terms of safety, feasibility and Minimal Residue Disease (MRD) response improvement (137).

## Conclusions

The tumor microenvironment represents a key factor in the maintenance and metastasis of different type of tumors. Therefore, the comprehension of the interactions between the microenvironment and the tumor constitutes a fundamental element for the study of new promising therapies, considering also the fact that the cellular and molecular connections that are established within the microenvironment itself strongly influence neoplastic growth and dissemination.

Focusing on hematological malignancies, the ultimate goal in the characterization of the leukemic microenvironment, therefore of the niches, is the realization of new pharmacological therapies targeting the BM in connection with actual optimized protocols. This could be achieved by hitting pharmacologically the interactions between the malignant cells and the hematopoietic cell-intrinsic networks that define and influence the niche or the BM microenvironment, such as CXCL12/CXCR4, NOTCH, MEK/ERK or PI3K/AKT signaling networks, or the interactions between interleukins, essential for BM niche homeostasis and functionality.

The formation of new vessels is also important for the growth, progression and maintenance of leukemic cells. An extremely important modulator in tumor microenvironment is hypoxia. This condition triggers a process that leads to the development of new blood vessels, generally exploited by tumor tissues to grow and create metastasis. Indeed, exploiting the distinctive hypoxia aspects and the hypoxia-dependent component such as HIF-1α or the angiogenesis factor VEGF in the context of hematological neoplasms and, in this specific manuscript, in ALL, represents a promising challenge to improve the current treatment scenario, selecting the optimal drug doses to reduce side effects and overcoming drug resistance.

The complete characterization of the leukemic tumor microenvironment for the formulation of innovative and specific therapies has not yet reached the highest level of completeness. However, the rapid discovery of microenvironment-ALL interactions increasingly aims at relevant clinical advances.

Comprehensive strategies are therefore needed for the characterization of the leukemic tumor microenvironment and leukemogenesis, further expanding proteomic and genomic methodologies, imaging and sequencing technologies and immunotherapeutic approaches, which can then consequently broaden the spectrum for ALL cure, both in pediatric and in adult patients.

## Author Contributions

CS, GV and LMN: idealization, literature search, intellectual input, manuscript editing and writing; IC, CB and EC: literature search and writing initial version of the manuscript. All authors contributed to the article final revision and approved the submitted version.

## Funding

This work was supported by current research funds Fondo di Ateneo per la Ricerca Scientifica (FAR) to LMN (project number FAR2011738), and to CS (project number FAR2095814). Moreover, this work was also supported with funding from the European Union’s Horizon 2020 research and innovation programme under the Marie Skłodowska-Curie grant agreement No. 895887, awarded to GV.

## Conflict of Interest

The authors declare that the research was conducted in the absence of any commercial or financial relationships that could be construed as a potential conflict of interest.
